# Endoplasmic reticulum stress response in spontaneously hypertensive rats is affected by myocardial ischemia reperfusion injury

**DOI:** 10.3892/etm.2014.2094

**Published:** 2014-11-27

**Authors:** XIAO-FU GUO, XIANG-JUN YANG

**Affiliations:** 1 Department of Cardiology, First Affiliated Hospital of Soochow University, Suzhou, Jiangsu 215006, P.R. China; 2 Department of Emergency and ICU, Suzhou Municipal Hospital (East Area), Suzhou, Jiangsu 215001, P.R. China

**Keywords:** endoplasmic reticulum, myocardial necrosis, glucose-regulated protein 78, PKR-like endoplasmic reticulum kinase, α-subunit of eukaryotic initiation factor 2, activating transcription factor 2

## Abstract

Cell apoptosis induced by endoplasmic reticulum (ER) stress appears to be one of the main causes of myocardial necrosis following myocardial ischemia/reperfusion (MI/R). The C/EBP homologous protein (CHOP) pathway is the main pathway through which apoptosis is induced during ER stress. Glucose-regulated protein 78 (GRP78) is an important protein involved in the CHOP pathway. The present study investigated the hypothesis that MI/R activates the CHOP pathway through signaling via a pathway involving PKR-like ER kinase (PERK), α-subunit of eukaryotic initiation factor 2 (eIF2α) and activating transcription factor 2 (ATF2). Immunohistochemical staining of the heart tissues from spontaneously hypersensitive rats indicated that MI/R injury increases CHOP and GPR78 protein expression levels. To further analyze the mechanism by which MI/R injury induces apoptosis by ER stress, the expression levels of five marker proteins involved in the hypothetical PERK-eIF2α-ATF2 pathway were detected, namely PERK, phosphorylated PERK (P-PERK), eIF2α, phosphorylated eIF2α (P-eIF2α) and ATF2. An increase in the collective expression levels of these proteins would indicate that apoptosis was induced by this signaling pathway. In addition, the study also explored whether hypertension affects the signaling pathway of MI/R-induced myocardial apoptosis by treating spontaneously hypertensive rats (SHRs) with captopril (an effective drug used to treat hypertension). Rats treated with captopril experienced a reduction in blood pressure to normal levels, but no marked differences in the expression levels of the tested proteins or in MI/R injury severity compared with those in untreated rats. These results suggest that MI/R activates the CHOP pathway during ER stress by activating the PERK-eIF2α-ATF2 pathway and that hypertension does not affect this signaling pathway.

## Introduction

Myocardial ischemia/reperfusion (MI/R) injury is an injury that is induced when blood returns to transiently ischemic myocardial tissues. It is associated with microvascular dysfunction including impaired endothelium-dependent dilation in arterioles, enhanced fluid filtration and leukocyte plugging in capillaries (1). MI/R is a complicated pathological process, the mechanism of which remains unclear. MI/R injury poses great harm to patients. It may lead to a decline in cardiac function and necrosis of myocardial tissue in ischemic areas, particularly in hypertensive patients (2). Furthermore, endoplasmic reticulum (ER) stress is coupled with MI/R injury. ER stress is a process in which the abnormal accumulation of unfolded and misfolded proteins in the ER damages ER functions and induces a number of pathological processes. Apoptosis may be induced by ER stress as a method of clearing damaged cells. The C/EBP homologous protein (CHOP) pathway is the main pathway involved in regulating the apoptosis induced by ER stress (3). CHOP belongs to the C/EBP transcription factor family. ER stress induces the expression of CHOP and leads to cell apoptosis; however, the targets of CHOP remain unknown (4). Glucose-regulated protein 78 (GRP78) is a significant protein involved in the CHOP pathway. GRP78, also known as immunoglobulin heavy chain-binding protein (Bip), is an important glucose-regulated molecular chaperone (5). Thus, whether the CHOP pathway is induced may be determined through detecting the expression levels of CHOP and GRP78 proteins.

ER stress also activates a highly conserved unfolded protein response (UPR). This response enhances the ability of the ER to process and refold proteins, helping to remove damaged proteins from the ER and to maintain homeostasis in cells. The UPR is mainly mediated by three ER transmembrane proteins, which are PKR-like ER kinase (PERK), inositol-requiring enzyme 1 (IRE1) and activating transcription factor 6 (ATF6) (6,7). Overexpression of GRP78 is induced when an excess of misfolded and unfolded proteins accumulate in the ER, activating the expression of PERK1, IRE1 and ATF6 (8). The activated, phosphorylated form of PERK (P-PERK) widely suppresses the synthesis of functional proteins in cells by phosphorylating its target protein, the α-subunit of eukaryotic initiation factor 2 (eIF2α). This process promotes the translation of certain other mRNAs, such as activating transcription factor 2 (ATF2) (9). ATF2 is a transcription factor and a member of the leucine zipper family of DNA-binding proteins (10). ATF2 is essential for amino acid responsiveness during the induction of CHOP expression (11). Upon dissociation from GRP78, ATF6 is transported into the nucleus and digested into its 50kDa activated form by restriction enzymes (12). These activated peptides are transported into the nucleus and bind to the ER, which activates related ER chaperones, such as GRP78, GRP94, protein disulfide isomerase and certain transcription factors such as CHOP (12).

In the present study PERK, P-PERK, eIF2α, phosphorylated eIF2α (P-eIF2α) and activating transcription factor 2 (ATF2) were selected as five biochemical markers to investigate whether MI/R activates the expression of CHOP through a PERK-eIF2α-ATF2 pathway. The study also explored whether the induction pathway of ER stress was changed under the effects of hypertension.

## Materials and methods

### Animals

The current study was performed in adherence with Chinese National Regulations for the Administration of Affairs concerning Experimental Animals. The animal models used were 16 male spontaneously hypersensitive rats (SHRs), weighing between 300 and 400 g and aged between 6 and 8 months, provided by the Animal Center in South Campus of Suzhou University (Jiangsu, China). SHRs were assigned to four groups: Group 1, sham surgery group; group 2, myocardial ischemia reperfusion group; group 3, myocardial ischemia reperfusion + captopril (a drug treating hypertension) group; and group 4, sham + captopril group. Groups 1 and 4 were sham surgery control groups under conditions of hypertension and non-hypertension, respectively. Groups 2 and 3 were experimental groups that underwent MI/R injury under conditions of hypertension and non-hypertension respectively. SHRs were randomly divided into the four groups. Captopril at a dose of 40 mg/kg was administered orally once per day to the rats in groups 3 and 4 rats for 7 weeks to lower their blood pressure to a normal level. The blood pressure and body weights of the rats were measured prior to surgery to ensure that captopril eliminated the symptoms of hypertension without affecting other body condition parameters. During surgery, animal preparation and the MI/R process were performed as described previously (13). Monitoring of blood pressure and heart rate was carried out using a non-invasive blood pressure meter (BP-98A; Softron Co. Ltd, Tokyo, Japan). Monitoring was initiated from immediately prior to anesthesia and continued until the end of the experiment, when the animals were humanely sacrificed under anesthesia. Samples of heart tissue were collected immediately for further analysis, including hematoxylin and eosin (H&E) staining, immunohistochemical staining and western blotting.

### H&E staining

Fresh heart tissues were placed into Bouin solution (4% formaldehyde) for perfusion fixation. Following this, they were dehydrated using alcohol and vitrified in dimethylbenzene. Samples were embedded in paraffin, sectioned and stained with H&E (SBT10001; Sunteambio Biotechnology, Shanghai, China). H&E staining was conducted according to previously described methods (14).

### Immunohistochemical staining and statistical analysis

Immunohistochemical staining was also performed on the formaldehyde-fixed and paraffin-embedded tissue samples. CHOP antibody (1:200; #2895; Cell Signaling Technology, Inc., Danvers, MA, USA) and GRP78 antibody (1:200; ab21685; Abcam, Cambridge, MA, USA) were used respectively as the primary antibodies. Peroxidase-conjugated goat anti-rabbit IgG (1:500; 111-035-003; Jackson ImmunoResearch, West Grove, PA, USA) was the secondary antibody. The procedures of immunohistochemical staining were based on previous protocols (15). When observing the slides under a microscope, cell nuclei were colored purple-blue and positive products were tan or yellow. Three photographs of each slide were selected randomly and analyzed using Image-Pro Plus 6.0 image analysis software (Media Cybernetics, Inc., Rockville, MD, USA). The software calculated the area of the positive regions, integrated optical density (IOD), number (n) of positive cells and IOD/n. Based on the IOD/n of each group, the mean ± standard deviation (SD) was calculated. Statistical analysis was carried out using SPSS 17.0 software (SPSS, Inc., Chicago, IL, USA) and P-values were calculated to compare the differences among groups. When P<0.05, the difference was considered statistically significant.

### Western blotting and statistical analysis

The expression of five important proteins involved in the PERK-eIF2α-ATF2 pathway was examined in a western blot assay. The primary antibodies were against PERK (1:1,000; #3192; Cell Signaling Technology, Inc., CST), P-PERK (1:1,000; #3179; Cell Signaling Technology, Inc.), eIF2α (1:1,000; #2103; Cell Signaling Technology, Inc.), P-eIF2α (1:1,000; #9721; Cell Signaling Technology, Inc.) and ATF2 (1:1,000; #9226; Cell Signaling Technology, Inc.). The secondary antibody was horseradish peroxidase (HRP)-labeled anti-rabbit and mouse IgG (H+L), which is polyvalent. The internal reference was a GADPH antibody (1:5,000; KC-5G5; Kandchen Bio-tech Inc., Shanghai, China). The whole procedure was based on a previous protocol (16). Based on the intensity of the protein bands, IOD was calculated using Gel-Pro Analyzer 4 software (Media Cybernetics, Inc.) for statistical study. Data was analyzed using the SPSS 17.0 statistics software package. Quantitative data were presented as the mean ± SD, and qualitative data as a percentage. One-way analysis of variance (ANOVA) was applied to make comparisons within one group and ANOVA was used to make mean comparisons in groups. A two-sided test was used to check statistics.

## Results

### Establishment of animal models

Data on the body weights and blood pressures of rats prior to and following captopril administration and surgery are displayed in Tables I–III. No significant differences in body weight (P>0.05) were identified among the four groups of rats prior to captopril administration on day 0. Following captopril administration to the rats in groups 3 and 4, the body weights of all the rats were measured weekly until surgery on day 49. During this time, no significant differences (P>0.05) were identified among the four groups. The blood pressure parameters in Table II showed no significant differences among the groups prior to captopril administration (P>0.05). Sixty days following the initiation of captopril administration to the rats in groups 3 and 4, a significant difference was observed between the blood pressure values in the rats of groups 1 and 2 and those in groups 3 and 4, which is demonstrated in Table III.

### Injury caused by MI/R

**Figure 1:**
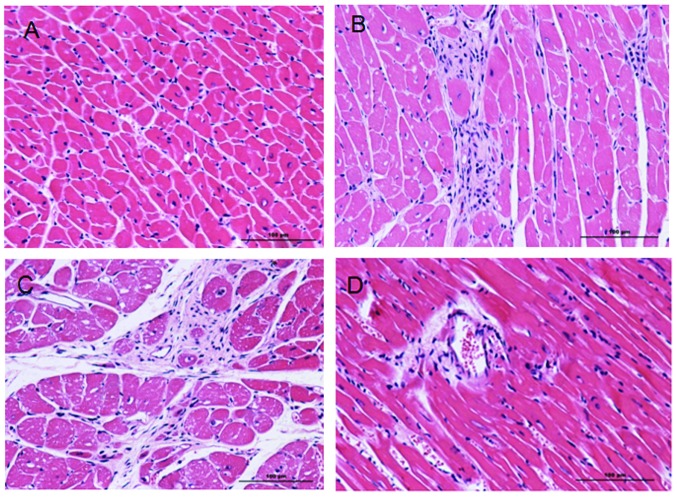
Hematoxylin and eosin (H&E) staining images of heart tissue samples from groups 1–4. H&E staining of (A) group 1, (B) group 2, (C) group 3 and (D) group 4. In the H&E staining images, the nucleus is blue and the cytoplasm is red. Collagen fibers show a varying red color. Comparing the experimental groups 2 and 3 with sham surgery groups 1 and 4 (B and C with A and D), severe injuries can be identified in the experimental groups, including obvious infarcts, inflammatory cell infiltration and the rupture and necrosis of myocardial cells that have lost their normal ordered structure. A large amount of fibrous scar tissue is visible in the infarction zone and infarcted border zone. Group 1, sham surgery; group 2, myocardial ischemia reperfusion; group 3, myocardial ischemia reperfusion and captopril treatment; group 4, sham surgery and captopril treatment.

Following H&E staining, the nuclei in the heart tissue were stained blue and cytoplasms were stained red. Collagen fibers were a varying red color. A comparison of the heart tissue from experimental groups 2 and 3 with that from sham surgery groups 1 and 4 (Fig. 1) revealed severe injuries in the experimental groups, including obvious infarcts, inflammatory cell infiltration and the rupture and necrosis of myocardial cells that had lost their normal ordered structure. Extensive fibrous scar tissue had formed in the infarction zone and infarcted border zone. Due to necrosis and injury in groups 2 and 3, it is evident that MI/R causes severe myocardial necrosis, regardless of hypertension.

### CHOP and GRP78 expression

**Figure 2:**
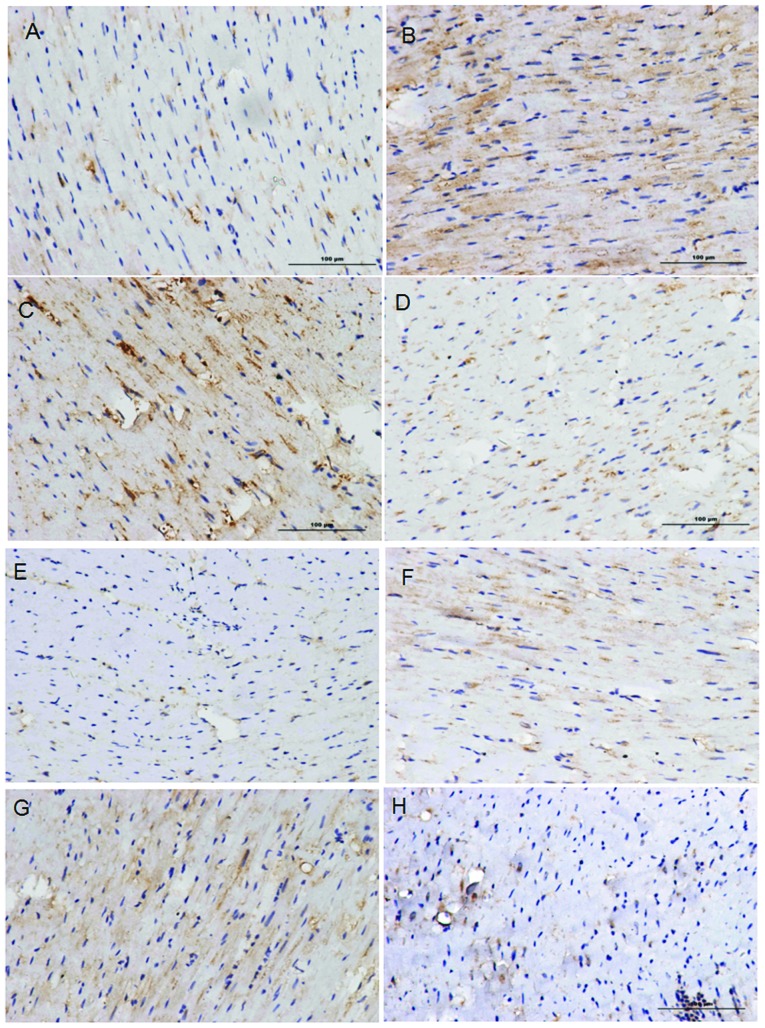
Immunohistochemical (IHC) staining of C/EBP homologous protein (CHOP) protein in heart tissue samples from (A) group 1, (B) group 2, (C) group 3 and (D) group 4. IHC staining of glucose-regulated protein 78 (GPR78) from (E) group 1, (F) group 2, (G) group 3 and (H) group 4. Cell nuclei are purple-blue in color and positive products are tan or yellow. The expression of CHOP and GRP78 in groups 2 and 3 is higher than in groups 1 and 4 which suggests that these proteins accumulate in areas where tissue injury and cell necrosis are the most severe. Group 1, sham surgery; group 2, myocardial ischemia reperfusion; group 3, myocardial ischemia reperfusion and captopril treatment; group 4, sham surgery and captopril treatment.

Statistical analysis of the images of the immunohistochemical staining (Fig. 2) was carried out using Image-Pro Plus 6.0 software. The expression levels of CHOP and GRP78 in groups 2 and 3 were higher than those in groups 1 and 4, thus suggesting that these proteins accumulate in areas where tissue injury and cell necrosis are the most severe. Comparison of the IODs for the CHOP-stained tissues (Table IV) revealed significant differences (P<0.05) between groups 1 and 3, and between groups 3 and 4. Comparison of the IODs for the GRP78-stained tissues revealed significant differences (P<0.05) between groups 1 and 2, and between groups 3 and 4 (Table V). No significant differences were identified among the other pairs. This comparison confirms that CHOP and GRP78 are involved in MI/R injury responses, indicating that MI/R injury induces ER stress through the CHOP pathway. The fact that no marked differences in the expression levels of CHOP or GRP78 were observed between groups 2 and 3 indicates that hypertension does not have a significant impact on ER stress induced by the CHOP pathway.

### Expression of five important proteins involved in the CHOP pathway

**Figure 3:**
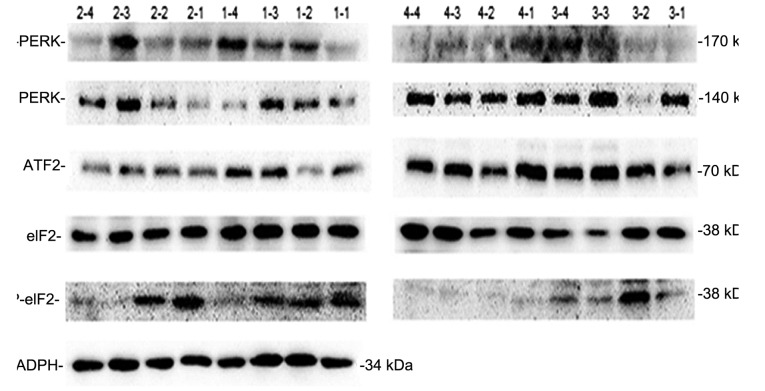
Images of the western blotting of PKR-like ER kinase (PERK), phosphorylated PERK (P-PERK), α-subunit of eukaryotic initiation factor 2 (eIF2α), P-eIF2α and activating transcription factor 2 (ATF2) proteins from the 16 samples. The numbers above the lanes indicate the specific rat tested, where the first number is the number of the group, and the second number is the number of the rat. Group 1, sham surgery; group 2, myocardial ischemia reperfusion; group 3, myocardial ischemia reperfusion and captopril treatment; group 4, sham surgery and captopril treatment.

As aforementioned, PERK, P-PERK, eIF2α, p-eIF2α and ATF2 were selected as five biochemical markers to investigate whether MI/R activates the expression of CHOP through a hypothetical PERK-eIF2α-ATF2 pathway. An SDS-PAGE image from the western blot analysis (Fig. 3) was processed using Gel-Pro Analyzer 4 software (data not shown). This revealed that the expression level of the five signaling proteins was higher as a whole in groups 2 and 3, than in groups 1 and 4. Although the differences on an average level were not marked, due to poor parallelism in the same group; this does not affect the conclusion that the PERK-eIF2α-ATF2 pathway was induced by MI/R injury.

No clear differences were observed between groups 2 and 3, indicating that hypertension is unlikely to affect the PERK-eIF2α-ATF2 pathway as the main pathway inducing ER stress.

## Discussion

According to World Health Organization (WHO) statistics, ischemic heart disease ranks as the greatest cause of mortality in humans. The most effective method of treating ischemic heart disease is myocardial reperfusion, using either thrombolytic therapy or primary percutaneous coronary intervention (PPCI) (17). However, the process of myocardial reperfusion may itself induce further cardiomyocyte death, a phenomenon known as myocardial ischemia/reperfusion (MI/R) injury (18–20). Although surgical techniques for the avoidance of MI/R are being developed, MI/R injury remains a severe problem.

The symptoms and severity of MI/R injury depend on numerous factors, including age, gender, duration and hypertension. The present study investigated the impact of hypertension on MI/R injury. The spontaneous hypertension rat (SHR), which has a syndrome similar to human spontaneous hypertension, was used as an animal model. This was considered appropriate for the investigation of MI/R injury combined with the factor of hypertension. Due to differences in genetic background and health conditions, common non-hypertensive rats were not suitable for use as controls. Thus, the corresponding control group was constructed by the administration of captopril to the SHR rats, so that their blood pressure was reduced to normal levels. According to current findings, captopril is a hypertension inhibitor that does not interfere with the signaling pathway under investigation in the present study. Captopril works either directly, by inhibiting the angiotensin converting enzyme (ACE), which blocks the renin angiotensin aldosterone (RAA) system, or indirectly, by inhibiting the sympathetic nervous system (SNS) at different levels (21). The 16 male SHRs were randomly divided into four groups. According to the weight and blood pressure measurements taken prior to surgery (Tables I and II), no significant differences existed among the groups. On day 60 after the initiation of drug administration, the blood pressures in the groups that were administered captopril had returned to normal (Table III) and were markedly lower than those in the SHR groups. In this way, experimental groups 1 and 2 of hypertensive rats and control groups 3 and 4 of non-hypertensive rats were successfully constructed. Following this, the rats in groups 2 and 3 underwent surgery to construct the MI/R experimental animal model group.

H&E staining verified the successful construction of the animal model (Fig. 1). In sham surgery groups 1 and 4, the area of necrosis and number of apoptotic cells was lower than in groups 2 and 3. ER stress, coupled with MI/R injury, usually results in the excessive accumulation of unfolded and misfolded proteins in the ER, which induces the unfolded protein response (UPR). Thus, signaling molecules involved in the UPR are typically used to indicate whether ER stress occurs. Under conditions of no ER stress, GRP78 binds to three transmembrane proteins, namely PERK, IRE1 and ATF6, to maintain the inactivated status of signal transduction factors. When the UPR process is activated, the activated IRE1α promotes the expression of UPR target molecules, which include endoplasmic reticulum stress responsive elements (ERSE) such as GRP78, to protect and recover homeostasis by reducing or terminating ER stress reactions. A number of studies have found that ER stress modulates not only the expression of apoptosis-inducing molecules such as CHOP and caspase-12, but also the expression/activation of certain survival molecules such as the growth arrest and DNA damage-inducible protein GADD34 and GRP78. Therefore, in the current study, one example of an apoptosis-inducing molecule and one of a survival molecule, namely CHOP and GRP78, were selected as markers to verify the induction of the ER stress regulatory pathway subsequent to MI/R injury.

Immunohistochemical staining results revealed the expression levels of CHOP and GRP78. When directly observing the images (Fig. 2), it is clear that the expression levels of the CHOP and GPR78 proteins in the MI/R injury experimental groups (groups 2 and 3) were higher than those in the sham surgery groups (groups 1 and 4). In addition, the expression of these proteins was concentrated in certain areas, where tissue necrosis and cell injury was the most severe. According to analysis of IOD values, CHOP immunohistochemical staining revealed that differences existed only between groups 1 and 3 and between groups 3 and 4, and the GRP78 staining revealed that differences existed only between groups 1 and 2 and between groups 3 and 4. These results confirm that MI/R injury induces ER stress through the CHOP pathway.

Following this, five signal proteins involved in the CHOP and GRP78 signaling pathway were selected for analysis to further explore whether MI/R injury induces the expression of CHOP through a PERK-eIF2α-ATF2 pathway. These proteins were PERK, P-PERK, eIF2α, P-eIF2α and ATF2,

Western blot results revealed that, overall, the expression levels of these five signal proteins were higher in groups 2 and 3 than in groups 1 and 4, although this difference was not evident at an average level due to a lack of parallelism. These results indicate that MI/R injury induces the expression of CHOP through a PERK-eIF2α-ATF2 pathway. A lack of parallelism existed in the western blot experiment results, which resulted in the differences exhibited being unclear. As an example, in the detection of PERK in group 3, a high expression level of PERK was detected in samples 3–1, 3–3 and 3–4, but no PERK was detected in sample 3–2. This may be due to the sampling location used in each rat, as only some of the cells in the necrotic tissue would undergo severe ER stress. In addition, individual differences among the rats would also be a major concern.

Hypertension did not demonstrate a significant impact on MI/R injury in the present study. Data analysis did not reveal any clear differences in protein expression levels between the rats in groups 2 and 3 in all experimental results, including those from H&E staining, immunohistochemical staining and western blotting. This indicates that under hypertension conditions, the main signaling pathway of MI/R injury does not change The PERK-eIF2α-ATF2 pathway remains the main pathway by which MI/R injury induces the expression of CHOP.

## Figures and Tables

**Table I:** Body weight (g) of rats following captopril administration and prior to surgery.

	Day							
	
Group	0	7	14	21	28	35	42	49
Group 1								
Rat 1	336.0	345.0	351.5	365.0	372.0	372.0	382.0	388.0
Rat 2	312.0	319.0	323.5	333.5	339.0	338.0	341.0	349.0
Rat 3	310.0	318.0	325.0	337.0	340.0	348.0	353.0	355.0
Rat 4	319.0	323.0	329.0	341.0	346.0	349.0	350.0	356.0
Group 2								
Rat 1	306.0	307.0	310.5	319.0	326.0	334.0	338.0	342.0
Rat 2	301.0	317.0	323.0	333.0	336.0	342.0	342.0	343.5
Rat 3	315.0	329.0	335.0	342.0	350.0	355.0	360.0	363.0
Rat 4	343.0	352.0	366.5	376.0	380.0	384.0	392.0	390.5
Group 3								
Rat 1	329.0	335.0	347.0	351.5	345.0	345.0	365.0	363.0
Rat 2	301.0	303.0	309.0	285.0	307.5	316.0	328.0	331.0
Rat 3	285.0	288.0	294.5	304.0	309.0	318.5	325.0	329.0
Rat 4	356.0	365.0	367.0	383.5	386.0	392.0	395.0	397.0
Group 4								
Rat 1	357.0	358.5	366.5	372.0	383.0	390.0	394.0	396.0
Rat 2	345.0	349.0	356.5	362.0	372.0	382.0	388.0	388.5
Rat 3	304.0	305.0	306.0	305.0	307.5	310.0	320.0	318.5
Rat 4	353.0	361.0	369.0	379.0	387.0	391.0	396.0	397.0

Group 1, sham surgery; group 2, myocardial ischemia reperfusion; group 3, myocardial ischemia reperfusion and captopril treatment; group 4, sham surgery and captopril treatment.

**Table II:** Blood pressure parameters of rats prior to captopril administration (day 0).

Group.	SBP	HR	MBP	DBP
Group 1				
Rat 1	213,221,241	426,417,427	172,178,212	152,157,198
Rat 2	142,156,183	475,467,481	125,138,176	117,129,173
Rat 3	149,160,150	409,420,387	123,123,118	120,105,102
Rat 4	173,210,192	372,354,337	150,185,166	139,172,153
Group 2				
Rat 1	233,236,242	384,389,415	181,180,213	155,152,199
Rat 2	166,186,181	405,408,465	150,152,147	142,135,130
Rat 3	158,166,160	418,399,392	143,129,124	136,110,106
Rat 4	179,178,144	376,461,428	143,142,126	125,124,117
Group 3				
Rat 1	179,210,205	389,378,359	159,167,177	149,145,163
Rat 2	169,172,162	421,398,382	140,136,121	126,118,101
Rat 3	148,148,160	490,466,463	124,118,129	112,103,114
Rat 4	192,221,172	428,457,450	159,181,138	143,161,121
Group 4				
Rat 1	212,160,192	489,476,498	185,132,162	172,118,147
Rat 2	191,163,177	488,478,434	157,132,152	140,117,140
Rat 3	196,142,155	356,360,378	161,129,129	143,123,115
Rat 4	193,223,236	485,460,478	148,181,185	126,160,160

SBP, systolic blood pressure; HR, heart rate; MBP, mean blood pressure; DBP, diastolic blood pressure. Group 1, sham surgery; group 2, myocardial ischemia reperfusion; group 3, myocardial ischemia reperfusion and captopril treatment; group 4, sham surgery and captopril treatment.

**Table III:** Blood pressure parameters of rats 60 days after the initiation of captopril treatment, according to group assignment.

Group	SBP	HR	MBP	DBP
Group 1				
Rat 1	211,199,208	399,412,413	176,181,188	159,172,178
Rat 2	157,152,154	386,382,382	133,129,133	121,118,123
Rat 3	197,193,202	290,288,297	173,166,170	161,153,153
Rat 4	171,170,174	303,301,302	135,130,130	117,110,108
Group 2				
Rat 1	185,183,173	411,410,403	157,163,148	143,153,136
Rat 2	169,172,168	436,403,401	149,151,144	139,141,132
Rat 3	171,164,180	407,382,389	134,127,139	116,108,119
Rat 4	180,171,170	473,459,470	145,133,136	128,114,119
Group 3				
Rat 1	118,115,120	483,495,472	106,102,108	100,96,102
Rat 2	145,149,148	324,314,304	127,115,122	118,97,109
Rat 3	144,139,142	399,388,390	106,113,109	87,100,93
Rat 4	149,138,145	330,346,331	128,121,111	118,113,94
Group 4				
Rat 1	156,139,130	321,338,369	126,107,110	111,91,100
Rat 2	123,128,115	337,331,353	103,100,91	93,86,79
Rat 3	126,136,136	354,370,359	103,120,109	92,112,96
Rat 4	153,149,154	434,446,426	119,112,127	102,94,114

SBP, systolic blood pressure; HR, heart rate; MBP, mean blood pressure; DBP, diastolic blood pressure. Group 1, sham surgery; group 2, myocardial ischemia reperfusion; group 3, myocardial ischemia reperfusion and captopril treatment; group 4, sham surgery and captopril treatment.

**Table IV:** P-values for comparisons of integrated optical density (IOD) values for C/EBP homologous protein (CHOP) staining between groups.

Group No.	1	2	3	4
1		0.116	0.007	0.539
2	0.116		0.148	0.310
3	0.007	0.148		0.023
4	0.539	0.310	0.023	

Group 1, sham surgery; group 2, myocardial ischemia reperfusion; group 3, myocardial ischemia reperfusion and captopril treatment; group 4, sham surgery and captopril treatment.

**Table V:** P-value for comparisons of integrated optical density (IOD) for glucose-regulated protein78 (GRP78) staining between groups.

Group No.	1	2	3	4
1		0.037	0.187	0.858
2	0.037		0.069	0.052
3	0.187	0.069		0.025
4	0.858	0.052	0.025	

Group 1, sham surgery; group 2, myocardial ischemia reperfusion; group 3, myocardial ischemia reperfusion and captopril treatment; group 4, sham surgery and captopril treatment.
